# Non-tip and rotatable sphincterotome for biliary cannulation in patients with Roux-en-Y gastrectomy

**DOI:** 10.1055/a-2239-2558

**Published:** 2024-02-02

**Authors:** Haruo Miwa, Kazuya Sugimori, Kazuki Endo, Ritsuko Oishi, Hiromi Tsuchiya, Takashi Kaneko, Shin Maeda

**Affiliations:** 1 Gastroenterological Center, Yokohama City University Medical Center, Yokohama, Japan; 2 Department of Gastroenterology, Yokohama City University Graduate School of Medicine, Yokohama, Japan

Balloon enteroscopy-assisted endoscopic retrograde cholangiopancreatography (BE-ERCP) has become widely used for patients with Roux-en-Y gastrectomy; however, selective biliary cannulation is still challenging 1 2 . A non-tip or rotatable sphincterotome has been reported to be beneficial in difficult cases 3 4 5 . A novel sphincterotome, Seeking Tome Zero (MTW Endoskopie Manufaktur, Wesel, Germany) ( Fig. 1 , Fig. 2 ), combines these features. Herein, we describe two successful cases with Roux-en-Y gastrectomy ( Video 1 ).

**Fig. 1:**
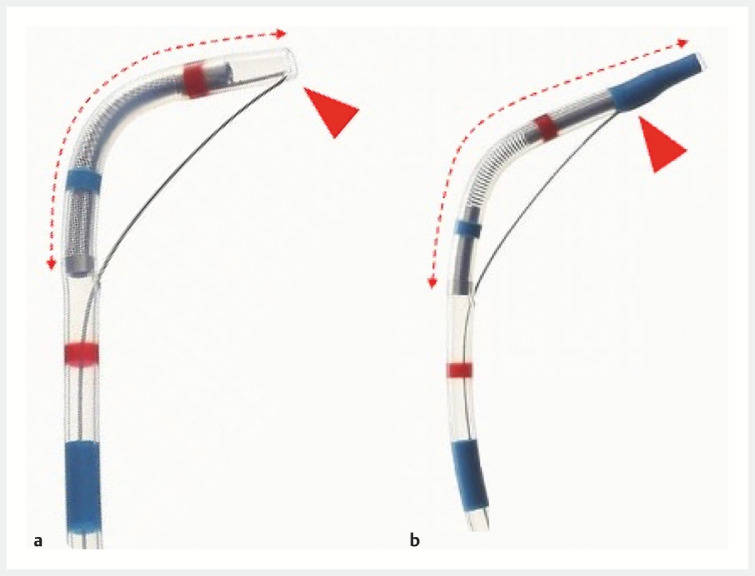
Seeking Tome Zero (MTW Endoskopie Manufaktur, Wesel, Germany) has no tip and a short angled part ( **a** ) compared with a normal endoscopic retrograde cholangiopancreatography catheter ( **b** ). Source: Abis Inc, Hyogo, Japan.

**Fig. 2:**
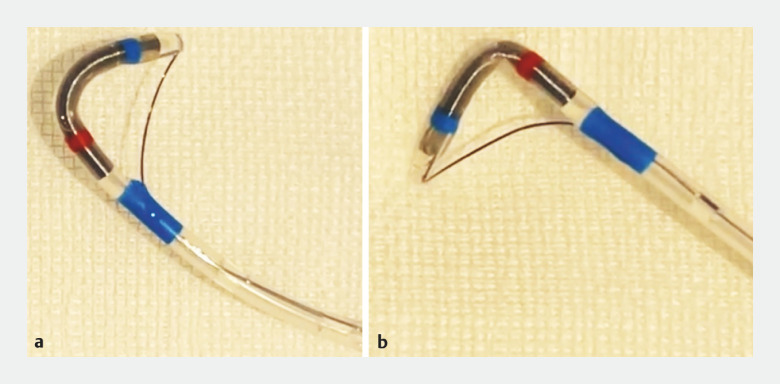
Seeking Tome Zero is easily rotated from one side ( **a** ) to the opposite side ( **b** ).

A non-tip sphincterotome facilitated changing the angle along a short distance and its rotatability allowed adjustment to the direction of the bile duct.Video 1

Case 1. An 87-year-old woman who had undergone Roux-en-Y gastrectomy was admitted to our hospital because of symptomatic choledocholithiasis. We attempted BE-ERCP using short-type single-balloon enteroscopy (SIF-H290S; Olympus Medical Systems, Tokyo, Japan). As biliary cannulation using a standard ERCP catheter was difficult due to a long and bent narrow distal segment, the catheter was substituted with Seeking Tome Zero ( Fig. 3 ). Following guidewire insertion into the pancreatic duct, a double guidewire technique was adopted. The sphincterotome was bendable at a short distance from the papilla, and a guidewire was successfully advanced into the bile duct ( Fig. 4 ).

**Fig. 3:**
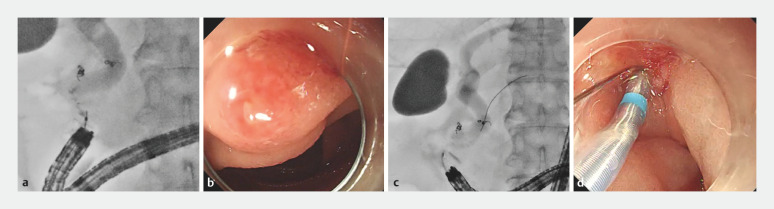
Case 1. **a** Cholangiography showed a long and bent narrow distal segment. **b** Biliary cannulation was started close to the papilla. **c,d** A guidewire was inserted into the main pancreatic duct.

**Fig. 4:**
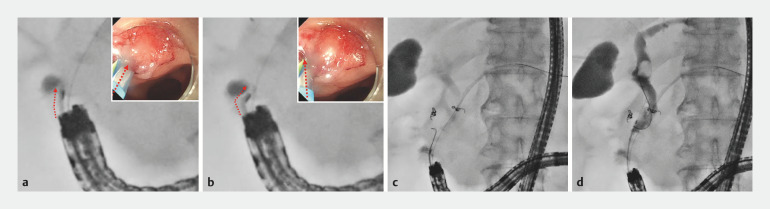
Case 1. **a,b** Seeking Tome Zero (MTW Endoskopie Manufaktur, Wesel, Germany) was bendable at a short distance from the papilla. **c,d** Biliary cannulation was achieved by gentle guidewire manipulation.

Case 2. A 64-year-old man who had undergone Roux-en-Y gastrectomy was admitted to our hospital because of asymptomatic choledocholithiasis. Despite the guidewire being advanced into the main pancreatic duct utilizing Seeking Tome Zero in BE-ERCP, the bile duct was oriented in the opposite direction. By rotating the handle, the sphincterotome was smoothly reversed in the direction of the bile duct. Following slight upward manipulation, the bile duct was aligned with the direction of the sphincterotome. Finally, biliary cannulation was achieved with gentle guidewire manipulation ( Fig. 5 ).

**Fig. 5:**
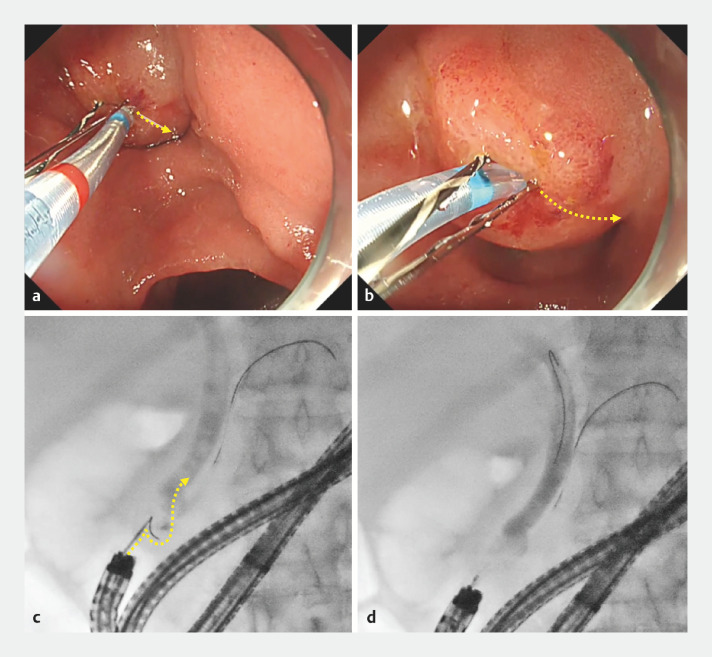
Case 2. The double-guidewire technique was performed with Seeking Tome Zero (MTW Endoskopie Manufaktur, Wesel, Germany). **a** The bile duct was oriented in the opposite direction to the sphincterotome. **b** Seeking Tome Zero was smoothly rotated in the direction of the bile duct. **c** Cholangiography showed a long and tortuous narrow distal segment. **d** Biliary cannulation was achieved.

To the best of our knowledge, this is the first report of biliary cannulation using Seeking Tome Zero for patients with altered anatomy. A non-tip sphincterotome facilitated changing the angle along a short distance and its smooth rotatability allowed adjustment to the axis of the bile duct.

Endoscopy_UCTN_Code_TTT_1AR_2AB

## Notes

Endoscopy E-Videos https://eref.thieme.de/e-videos
*E-Videos* is an open access online section of the journal *Endoscopy* , reporting on interesting cases and new techniques in gastroenterological endoscopy. All papers include a high-quality video and are published with a Creative Commons CC-BY license. Endoscopy E-Videos qualify for HINARI discounts and waivers and eligibility is automatically checked during the submission process. We grant 100% waivers to articles whose corresponding authors are based in Group A countries and 50% waivers to those who are based in Group B countries as classified by Research4Life (see: https://www.research4life.org/access/eligibility/ ). This section has its own submission website at https://mc.manuscriptcentral.com/e-videos .
